# Penile surgery for patients with Peyronie’s disease initially treated with collagenase clostridium histolyticum or surgery: a claims database analysis

**DOI:** 10.1038/s41443-021-00522-8

**Published:** 2022-01-10

**Authors:** Landon Trost, Huan Huang, Xu Han, Chakkarin Burudpakdee, Yiqun Hu

**Affiliations:** 1 Mayo Clinic, Rochester, MN USA; 2 Male Fertility and Peyronie’s Clinic, Orem, UT USA; 3 IQVIA, Falls Church, VA USA; 4 Endo Pharmaceuticals Inc., Malvern, PA USA

**Keywords:** Sexual dysfunction, Translational research

## Abstract

Collagenase clostridium histolyticum (CCH) is an injectable therapy targeting collagen present in penile plaques in Peyronie’s disease (PD). Data comparing CCH to penile surgery are limited, and long-term therapeutic outcomes are unknown. This retrospective analysis used a US claims database (January 2014–June 2017) to determine the percentage of men with subsequent penile surgery among those who initially received CCH (*n* = 1227) or surgery (index treatment; *n* = 620) for PD. Eligible patients were aged ≥18 years with continuous enrollment ≥6 months before and ≥12 months after index treatment date. During 12 months of post-index treatment follow-up, fewer patients with PD initially treated with CCH (4.6% [56/1227]) had subsequent penile surgery versus those initially treated with penile surgery (10.3% [64/620]; *p* < 0.0001). Mean ± SD time to first subsequent surgery after initial PD treatment was longer in the CCH versus surgery cohort (7.7 ± 3.0 vs 1.7 ± 3.2 months). The likelihood of subsequent surgery varied by initial surgery type: 18.2% after plaque incision or excision with grafting; 11.6% after penile implant; and 8.2% after tunical plication. Patients with PD who received CCH first were less likely to undergo subsequent surgery compared with those who received surgery first within a 12-month post-treatment follow-up.

## Introduction

Peyronie’s disease (PD) is a wound-healing disorder characterized by the progressive accumulation of collagen-rich fibrotic plaques in the tunica albuginea [1, 2]. Penile plaque formation may cause penile deformity, pain, and sexual dysfunction, resulting in psychological distress for affected men and their partners [1, 3]. Treatment of PD has included surgical intervention, such as penile tunical plication; penile plaque incision or excision with grafting (I/E & G); and prosthesis implantation [4]. While these surgeries can be effective for reducing penile curvature caused by PD, they are associated with adverse events, such as perceived or actual penile length reduction, sensory changes, erectile dysfunction, and implant complications [5].

Collagenase clostridium histolyticum (CCH) is an injectable therapy that targets collagen present in penile plaques of patients with PD [6]. CCH was approved in 2013 by the US Food and Drug administration for the treatment of adult men with PD with a palpable plaque and curvature deformity of ≥30° [7]. The safety and efficacy of CCH for the treatment of men with PD have been demonstrated in randomized, placebo-controlled studies [8], prospective, open-label studies [3, 9], and a prospective, long-term (up to 5 years), observational study [10]. CCH is an effective, nonsurgical intervention for PD and has been increasingly used as a first-line therapy [11]. However, data comparing CCH to penile surgery in the clinic practice setting are limited, and the long-term outcomes of the therapeutic effect (i.e., need for revision or additional surgery) are unknown. Therefore, the objective of the current analysis was to evaluate the likelihood of subsequent surgery in patients newly diagnosed with PD who received CCH or penile surgery as initial treatment for PD.

## Materials and methods

This retrospective cohort analysis was conducted using data from the IQVIA Real-World Data Adjudicated Claims—US Database. This database includes adjudicated health plan claims for >150 million individuals, encompasses the majority of US hospitals and healthcare providers and is representative of the national commercially insured population. Information stored in the database is de-identified and includes patient diagnoses, as well as inpatient and outpatient procedures, prescriptions, and payments; data collection complies with the Health Insurance Portability and Accountability Act of 1996. Because the current retrospective analysis used deidentified data obtained from the database (i.e., a secondary source), approval by an institutional review board or ethics committee was not obtained.

Identification of patients with PD in the database was based on International Classification of Diseases, Ninth/Tenth Revision, Clinical Modification diagnosis codes for PD (607.85) or induration penis plastica (N48.6). The index treatment date was designated as the first claim for CCH (based on National Drug Code [66887000301, 66887000302] or Healthcare Common Procedure Coding System [J0775]) or penile surgery (based on International Classification of Diseases, Ninth/Tenth Revision, Clinical Modification procedure codes for tunical plication [0VQS0ZZ, 64.49, 0VQS3ZZ, 0VQS4ZZ, 0VQSXZZ], penile plaque incision or excision [0VCSXZZ, 0VCS3ZZ, 0VCS0ZZ, 0VBSXZZ, 0VBS0ZZ, 0VBS3ZZ, 64.92, 64.2] or penile prosthesis implantation [i.e., penile implant; 0VUS0JZ; 0VUS4JZ, 0VUSXJZ, 64.95, 64.97]).

Adults aged ≥18 years with a diagnosis of PD who received CCH injection or penile surgery between January 1, 2014, and June 30, 2017, were eligible for inclusion in the current analysis. The date of the first CCH injection claim (CCH cohort) or first penile surgery claim (surgery cohort) was defined as the index treatment date. Patients were also required to have ≥6 months of continuous health plan enrollment before the index treatment date (baseline period) and ≥12 months of continuous enrollment after the index treatment date. Penile surgeries were identified post-index for 12 months as well as 24 months (exploratory analysis). Comparisons between the CCH and surgery cohorts with regard to baseline demographics and clinical characteristics were performed using parametric *t*-tests (for means) and Wilcoxon rank-sum tests (for medians) for continuous variables, or chi-square tests for categorical variables. Subsequent post-index surgeries between the two cohorts were compared using chi-square tests. Analyses were performed using SAS^®^ version 9.4 software (SAS Institute, Inc., Cary, NC), assuming a two-tailed test of significance and type I error rate of 0.05.

## Results

**Fig. 1:**
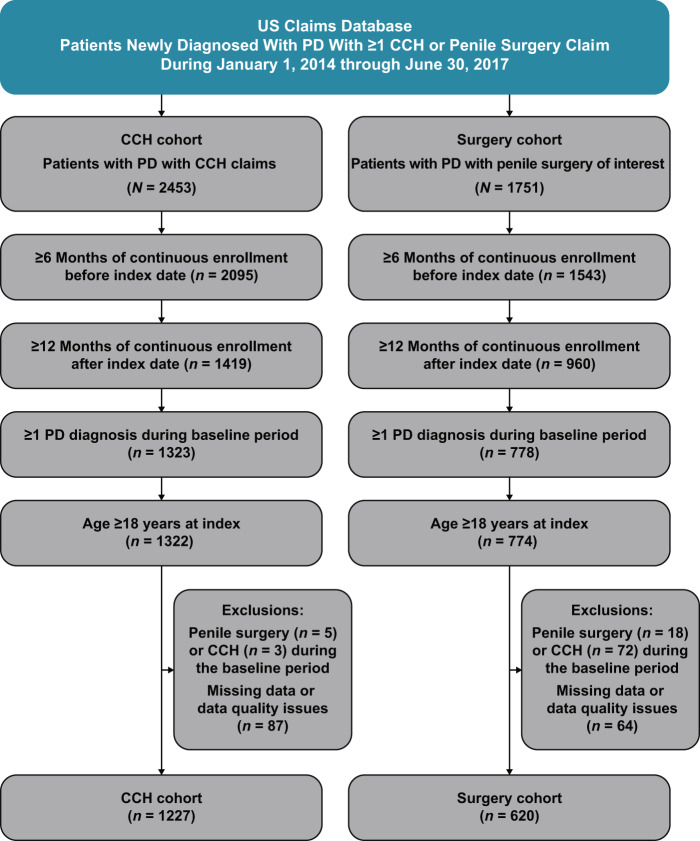
Study flow. CCH collagenase clostridium histolyticum, PD Peyronie’s disease. Figure adapted from Trost et al. Sex Med. 2021;9(2):100321 [12], via a Creative Commons CC BY 4.0 open access license.

A total of 4204 patients with PD filed ≥1 claim for CCH (*n* = 2453) or surgery (*n* = 1751) during the 3.5-year period. Of these patients, 1227 in the CCH cohort and 620 in the penile surgery cohort met all study criteria and were included in the analysis (Fig. 1) [12]. As previously reported, there were some significant differences in patient characteristics observed between the two cohorts: the surgery cohort had a higher mean Charlson Comorbidity Index score, as well as higher rates of prior prostatectomy, comorbid erectile dysfunction, and comorbid penile pain versus the CCH cohort (Table 1) [12]. The mean ± standard deviation (SD) and median time from first PD diagnosis during the baseline period to index treatment were similar for the CCH and surgery cohorts. In a subanalysis of 1044 CCH cohort patients with available data on all CCH injections received, the mean ± SD number of CCH injections was 6.1 ± 3.0, with 32.6% (340/1044) of patients receiving 8 CCH injections (i.e., presumably completing a full treatment course [four treatment cycles, each consisting of two injection procedures], as noted in the US prescribing information [7]), and 18.6% (194/1044) receiving 6 CCH injections. Among the 620 patients with penile surgery as the initial treatment, tunical plication was the most common surgical procedure (*n* = 220 [35.5%]), followed by penile implant (*n* = 172 [27.7%]); combination surgery (e.g., tunical plication and penile implant; *n* = 151 [24.4%]); and plaque I/E & G (*n* = 77 [12.4%]).

**Table 1:** Patient demographics and clinical characteristics.

Parameter	CCH cohort (*n* = 1227)	Surgery cohort (*n* = 620)	*p* value
Mean age, (SD), y	54.7 (7.3)	54.2 (9.0)	0.16
Range	18–72	18–72	
Insurance type, *n* (%)			
Commercial	819 (66.7)	377 (60.8)	0.001
Medicaid	8 (0.7)	17 (2.7)	
Medicare	4 (0.3)	3 (0.5)	
Self-insured	383 (31.2)	220 (35.5)	
Other/unknown	13 (1.1)	3 (0.5)	
Region, *n* (%)			
Northeast	180 (14.7)	95 (15.3)	0.95
Midwest	301 (24.5)	152 (24.5)	
South	624 (50.9)	316 (51.0)	
West	122 (9.9)	57 (9.2)	
Mean Charlson Comorbidity Index (SD)	1.1 (1.7)	1.5 (2.0)	<0.0001
Range	0–15	0–14	
Baseline comorbidities of interest, *n* (%)			
Corporeal rupture	10 (0.8)	11 (1.8)	0.07
Erectile dysfunction	632 (51.5)	430 (69.4)	<0.0001
Penile pain	142 (11.6)	126 (20.3)	<0.0001
Medical history of interest, *n* (%)			
Diabetes	264 (21.5)	157 (25.3)	0.07
Penile trauma	10 (0.8)	11 (1.8)	0.07
Prostatectomy	12 (1.0)	32 (5.2)	<0.0001
Time from PD diagnosis to index treatment, mean (SD), months	11.9 (12.7)	11.6 (13.3)	0.72

Table adapted from Trost et al. Sex Med. 2021;9:100321 [12], via a Creative Commons CC BY 4.0 open access license.

*SD* standard deviation.

**Fig. 2:**
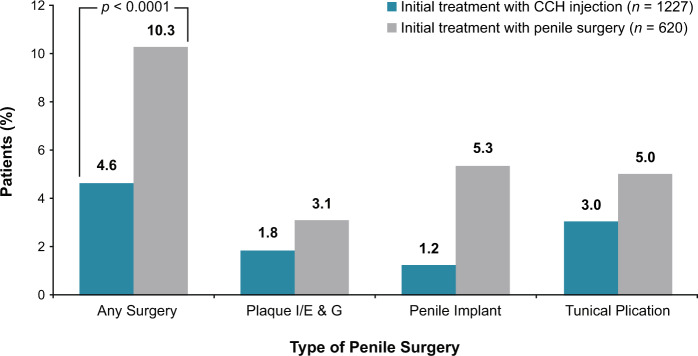
Percentage of patients with penile surgery within 12 months after initial treatment with CCH or penile surgery (index treatment) for the cohorts overall and by subsequent penile surgery type^a^. ^a^Patients may have had ≥1 post-index penile surgery. *P* value from chi-square test. CCH collagenase clostridium histolyticum, I/E & G incision or excision with grafting.

During the 12 months after the index treatment date, significantly fewer patients treated with CCH as initial PD therapy underwent subsequent penile surgery compared with patients who received surgery as initial therapy (*p* < 0.0001; Fig. 2; Table 2). The mean ± SD time to subsequent surgery was 7.7 ± 3.0 months for the CCH cohort versus 1.7 ± 3.2 months for the surgery cohort. Among the 56 (4.6%) patients who had ≥1 penile surgery during the 12-month follow-up period after the initial CCH treatment, 66.1% (*n* = 37) had tunical plication, 39.3% (*n* = 22) had plaque I/E & G, and 26.8% (*n* = 15) had a penile implant (some patients underwent ≥1 post-index penile surgery). The mean time from CCH treatment to any subsequent penile surgeries in these patients was similar (7–8 months), regardless of surgery type(s).

**Table 2:** Percentage of patients who underwent subsequent penile surgery within 12 and 24 months of index treatment date^a^.

Subsequent surgery	12-Month follow-up, *n* (%)		24-Month follow-up, *n* (%)	
	CCH (*n* = 1227)	Surgery (*n* = 620)	CCH (*n* = 630)	Surgery (*n* = 354)
Any surgery	56 (4.6)^b^	64 (10.3)	55 (8.7)^c^	38 (10.7)
Plaque I/E & G	22 (1.8)	19 (3.1)	20 (3.2)	9 (2.5)
Penile implant	15 (1.2)	33 (5.3)	14 (2.2)	16 (4.5)
Tunical plication	37 (3.0)	31 (5.0)	35 (5.6)	23 (6.5)

*I/E & G* incision or excision with grafting.

^a^Data reported for individuals with follow up data at 12 or 24 months after index date. Patients may have undergone ≥1 post-index penile surgery.

^b^*p* < 0.0001 vs surgery.

^c^*p* = 0.3 vs surgery.

The subsequent surgical rates and types received by the patients who had surgical procedure(s) as initial therapy varied based on the initial penile surgery type(s). Among all index surgery types, patients who received plaque I/E & G as the index procedure had the highest rate of subsequent (i.e., additional) surgery within the 12-month follow-up period (Table 3). Men initially treated with plaque I/E &G had a ~6-times higher rate of subsequent penile implant compared with men initially treated with plication alone (Table 3). Among patients who received penile implant surgeries, 20 (11.6%) needed subsequent surgeries. Of these men, all 20 (100%) received a repeat penile implant, with 5 (25.0%) undergoing concomitant plication and 3 (15.0%) with concomitant I/E & G (Table 3). Three (0.5%) of 620 patients in the surgery cohort were treated with CCH within 12 months after the post-index treatment date (mean ± SD time from surgery to CCH, 4.5 ± 2.7 months).

**Table 3:** Percentage of patients in penile surgery cohort who underwent subsequent penile surgery within 12 months of index treatment date.

Initial (index) penile surgery type	Subsequent surgery, *n* (%)^a^			
	Any surgery	Plaque I/E & G	Penile implant	Tunical plication
Total surgery cohort (*n* = 620)	64 (10.3)	19 (3.1)	33 (5.3)	31 (5.0)
Tunical plication (*n* = 220)	18 (8.2)	4 (1.8)	2 (0.9)	12 (5.5)
Penile implant (*n* = 172)	20 (11.6)	3 (1.7)	20 (11.6)	5 (2.9)
Plaque I/E & G (*n* = 77)	14 (18.2)	8 (10.4)	4 (5.2)	8 (10.4)
Combination surgery (*n* = 151)^b^	12 (7.9)	4 (2.6)	7 (4.6)	6 (4.0)

*I/E & G* incision or excision with grafting.

^a^Patients may have undergone ≥1 post-index penile surgery.

^b^Underwent ≥1 surgery (i.e., combination surgery) as index treatment*.*

For patients with follow-up data for 24 months after the index date, the percentage of patients with subsequent surgery remained lower in the CCH cohort (8.7% [55/630]) compared with the surgery cohort (10.7% [38/354]), although the difference was not statistically significant (*p* = 0.30; Table 2).

## Discussion

The current US claims database analysis found that the rate of subsequent PD surgery within 12 months was significantly lower for patients with PD who had been treated with CCH initially, compared with those who received penile surgery as the index treatment (*p* < 0.0001). Although the rate of subsequent PD surgery during 24 months of follow-up was numerically lower for patients initially treated with CCH versus surgery, the difference between cohorts was not statistically significant at this point in time. The findings of the current analysis are consistent with the low rates of post-CCH surgical interventions previously reported [13, 14]. A multicenter retrospective study of 918 patients with PD treated with CCH reported that 3.8% of patients overall, and 4.6% of those who received four treatment cycles, had subsequent surgery to correct residual penile curvature [13]. Similarly, a single-center retrospective study of 162 patients treated with CCH found that 6.2% required surgery to address persistent penile curvature and impaired erectile function [14]. Importantly, published data also indicate that prior CCH treatment for PD does not increase postoperative complications or negatively impact outcomes for patients receiving subsequent penile surgery [15–17]. This suggests that initial treatment with CCH will not interfere with subsequent surgery for PD, in the event that it is needed.

Tunical plication is the most common surgery employed for the treatment of PD [18], and data from the current analysis were consistent with this observation. In the surgery cohort, fewer patients initially treated with tunical plication had additional surgery than the other major surgery types within 12 months: subsequent surgery rate was 2.2 times lower than the plaque I/E & G subgroup and 1.4 times lower than the penile implant subgroup. Men initially treated with plaque I/E & G appeared to have a higher retreatment rate than those with other surgery types, including a ~6 times higher rate of subsequent penile implant compared with the initial tunical plication alone group during 12 months of follow-up. This is a clinically meaningful difference in the rate of subsequent penile implant and may be relevant when counseling patients about the possibility of erectile dysfunction after initial surgical treatment for PD. However, it should be noted that data on PD severity at the time of the index treatment, plaque location, surgeon experience, and patient preference—all of which would likely have influenced the type of initial surgery selected and the timing and selection of subsequent surgery—were not available for assessment in the current study. The rate of penile implant revision within 12 months (11.6% of men with penile implant as index surgery) was high. To the extent that this finding is reflective of actual re-intervention and revision rates, these data confirm that men with PD and erectile dysfunction may have complex reconstructive needs that are best addressed by a specialist in PD and prosthetic urology.

The selection of CCH or surgery as the initial treatment for PD depends on multiple factors, including a number of disease-related and patient-specific factors and patient and clinician preferences [19]. There is general consensus that penile surgery is indicated only after PD has stabilized and entered the chronic phase, in order to reduce the likelihood of deformity recurrence [20, 21]. Men with medication-resistant erectile dysfunction and PD may prefer surgical options that include a penile implant, in order to address both conditions [22]. In the current analysis, data suggested that patients with comorbid erectile dysfunction or penile pain, with a higher disease burden (i.e., Charlson Comorbidity Index), or who had a prostatectomy previously were more likely to receive surgery rather than CCH as initial therapy for PD. It was not the aim of this study to examine differences in PD treatment selection based on insurance type; and, as almost all patients were either in a commercial plan or self-insured, such comparisons were not feasible. Nonetheless, it is important to note that insurance coverage, rather than the best-individualized treatment strategy, may influence the choice of initial treatment in men with PD, which indicates a need for improved treatment access.

The published literature has suggested that patients with PD should wait at least 6 months after the last CCH injection or their initial surgery before undergoing subsequent penile surgery [16, 20]. This timeframe is suggested to allow for healing and assessment of fibrosis (e.g., occurrence of residual or new curvature deformity) and/or development of erectile dysfunction [16, 20]. In the current study, the meantime to subsequent surgery for the CCH cohort was consistent with this 6-month timeframe (7.7 months); however, for the surgery cohort, the meantime to subsequent surgery was substantially shorter (1.7 months). The longer duration to subsequent surgery with the CCH cohort may be related to the longer timeframe required to complete CCH treatment cycles (e.g., minimum of 24 weeks for 4 treatment cycles) [7].

Strengths of the current study include the evaluation of real-world treatment patterns using a large administrative claims database with broad generalizability for the United States. Limitations include the retrospective nature of the analysis, no randomization of treatment, and differences in baseline clinical characteristics between cohorts. In addition, the database lacked information on plaque location and PD severity (e.g., degree of penile curvature), and had limited inclusion of older patients (i.e., Medicare) and Medicaid beneficiaries. Given the limitations of the information available in the database, the extent to which baseline demographic and clinical characteristics differed between treatment cohorts could not be fully determined. Furthermore, data were not available on whether subsequent surgery, if required, was for the same penile plaque or a new one, or whether repeat IPP surgery was performed for the purposes of PD treatment or because of a device malfunction. Patient attributes contributing to the selection and type of subsequent surgery, in either initial treatment cohort, could not be extracted from the database. Finally, because it takes approximately 6 months to complete the entire four treatment cycles of CCH, and because it is generally recommended to have a waiting period after the last CCH injection before initiating secondary interventions, [16, 20] this analysis was vulnerable to a lead-time bias. This may have resulted in an underestimation of the incidence of surgical interventions after CCH treatment. However, this potential effect was partially mitigated through follow-up to the 24-month time point, which demonstrated persistently higher (although not statistically significantly different) rates of intervention in the non-CCH cohort.

In this US retrospective claims database analysis of patients with newly diagnosed PD, more men received treatment with CCH injection than all penile surgical therapies combined from January 2014 through June 2017. Men treated with CCH during the 12-month observation period underwent penile surgery at a lower rate compared with those initially treated with penile plication, plaque I/E & G, or penile implant. Men initially treated with plaque I/E & G appeared to have a higher retreatment rate than other surgery types, including a ~6 times higher rate of subsequent penile implant compared with the initial plication alone group during 12 months of follow-up. A small number of men initially treated with surgery subsequently received CCH. A prospective PD study is warranted to better characterize long-term outcomes, individual patient parameters, and prognostic factors for subsequent surgery.
